# Innate sensing of viral infection by pDCs and regulation by IFN-α and HMGB1 of TRAIL expression on pDCs and NK cells

**DOI:** 10.1186/1471-2334-14-S2-O8

**Published:** 2014-05-23

**Authors:** Héla Saïdi, Marlène Bras, Pauline Formaglio, Bruno Charbit, Jean-Philippe Herbeuval, Marie-Lise Gougeon

**Affiliations:** 1 Pasteur Institute, Paris, France

## Introduction

At an early stage of HIV-1 transmission, plasmacytoid dendritic cells (pDCs) and natural killer (NK) cells are recruited into mucosal tissues. pDCs are the major source of type I interferon (IFN-α), a powerful innate antiviral cytokine, and a strong pDC response is associated with spontaneous virus control. The contribution of pDCs to licensing NK cells and inducing antiviral immunity make them a key player in the early phase of HIV-1 infection. In contrast, the chronic expression of IFN-α was found to be a key mediator for HIV pathogenesis. We addressed herein the question of the molecular mechanisms involved in the generation of Interferon-producing Killer pDCs (IKpDCs), and the consequences on viral control.

## Materials and methods

pDCs and NK cells were negatively sorted from the blood of healthy donors with specific magnetic beads. NK cells were kept either in a resting state or activated with PMA/ionomycine for 2 hrs. pDCs were stimulated with ODB 2006, ODN 2216, or were infected with R5-HIV-1 at various concentrations. In some experiments, pDCs were cocultured with NK cells for 24 hrs at NK:DC ratio of 1:5. Phagocytosis experiments were performed with fluorescent microsphere beads. The mutual influence of NK-pDC interaction was analyzed by multiparametric flow cytometry, combining maturation and cell death/survival markers with cytokine detection, and released cytokines in cultures supernatants were quantified with the MAP luminex technology.

## Results

We report that high concentrations of HIV-1 induced both survival and maturation of pDCs. These pDCs were characterized by increased size and the expression of HLA-DR, CD40, CD86, CCR7 and CD83 molecules. This phenotypic maturation was coupled to a functional maturation since HIV-1-infected pDCs exhibited higher phagocytic activity, expressed mTRAIL and released high levels of pro-inflammatory cytokines and chemokines (IL-6, IL-8, TNF-α, IP-10, MIP-1α and MIP1-β). The release of these mediators was dependent on both IFN-α and HMGB1. Finally, pDC activation by HIV-1 triggered the expression of mTRAIL on NK cells. Interestingly, both IFN-α and HMGB1 were required to induce killer NK cells.

## Conclusion

We report for the first time the critical role of both HMGB1 and INF-α on the expression of TRAIL at the surface of both infected-pDCs and NK cells during their interaction, and also on the triggering of beta-chemokines synthesis by pDCs. These data suggest that the cross-talk of HIV-infected pDCs with NK cells favors the emergence of both killer pDC and cytotoxic NK cells that is essential for the control of viral replication at the early stage of the infection.

